# Health Professionals’ Knowledge of Probiotics: An International Survey

**DOI:** 10.3390/ijerph16173128

**Published:** 2019-08-28

**Authors:** Sabina Fijan, Anita Frauwallner, László Varga, Tomaž Langerholc, Irena Rogelj, Mateja Lorber, Peter Lewis, Petra Povalej Bržan

**Affiliations:** 1 Faculty of Health Sciences, University of Maribor, Žitna ulica 15, 2000 Maribor, Slovenia; 2 Institut Allergosan, Pharmazeutische Produkte Forschungs- und Vertriebs GmbH, Gmeinstrasse 13, 8055 Graz, Austria; 3 Department of Food Science, Faculty of Agricultural and Food Sciences, Széchenyi István University, Lucsony u. 15-17., 9200 Mosonmagyaróvár, Hungary; 4 Department of Microbiology, Biochemistry, Molecular Biology and Biotechnology, Faculty of Agriculture and Life Sciences, University of Maribor, Pivola 10, 2311 Hoče, Slovenia; 5 Biotechnical Faculty, University of Ljubljana, Jamnikarjeva 101, 1000 Ljubljana, Slovenija; 6 School of Nursing and Midwifery, University of Western Sydney, Locked Bag 1797, Penrith, NSW 2751, Australia; 7 Faculty of Medicine, University of Maribor, Taborska ulica 8, 2000 Maribor, Slovenia; 8 Faculty of Electrical Engineering and Computer Science, University of Maribor, Koroška cesta 46, 2000 Maribor, Slovenia

**Keywords:** probiotics, knowledge, health professionals

## Abstract

The objective of this study was to survey health professionals to investigate their knowledge of probiotics. An online survey was conducted to gather data on the knowledge of health professionals. The online survey was distributed via email and social media platforms using snowball sampling. A total of 1066 health professionals (859; 80.6% female) from 30 countries responded to the survey. Most of the respondents evaluated their knowledge of probiotics as medium (36.4%) or good (36.2%). Only 8.9% of the respondents rated it as excellent. No statistical difference in knowledge was found between male and female health professionals. Over 80% of pharmacists, allied health professionals, medical doctors and dentists, and other health professionals knew the correct definition of probiotics as “live microorganisms that, when administered in adequate amounts, confer a health benefit on the host”, whereas three quarters of registered nurses and midwives and less than two thirds of psychologists identified the correct definition. Statistically, more female than male health professionals knew the correct definition of probiotics. The most frequently recognized species of bacteria containing probiotic strains were *Lactobacillus acidophilus* (92%), *Bifidobacterium bifidum* (82%), and *Lactobacillus rhamnosus* (62%). The opinions on when it is best to take probiotics were different (χ^2^ = 28.375; p < 0.001), with 90.2% of respondents identifying that probiotics have beneficial effects if taken during antibiotic therapy, 83.5% for diarrhea, 70.6% for constipation, 63.3% before traveling abroad, and 60.4% for treating allergies. Almost 79% of health professionals involved in this study have advised their patients to use probiotics and 57.5% of the respondents wanted to learn more about probiotics. All things considered, health professionals have a medium level of knowledge of probiotics, which could be improved by the implementation of targeted learning programs. As probiotics have many beneficial effects in a wide range of health areas, health professionals need to adopt the use of probiotics in clinical practice.

## 1. Introduction

The well-known definition of probiotics was originally accepted in 2001 at an expert consultation of international scientists working on behalf of the Food and Agriculture Organization of the United Nations (FAO) and the World Health Organization (WHO) [1]. In 2002, a FAO/WHO working group produced guidelines to assist with the interpretation of the original document [2]. For almost two decades, the definition has been widely used in the scientific community. In 2014, the International Scientific Association for Probiotics and Prebiotics (ISAPP) published the most recent and widely accepted definition of probiotics as follows: “*live microorganisms that, when administered in adequate amounts, confer a health benefit on the host.*” [3].

Prior to the acceptance of this definition, only strain-specific probiotics were acknowledged to confer health benefits [4]; however, on the basis of a large number of well-designed clinical trials, it has been agreed that certain beneficial health effects of several strains of a number of well-studied microbial species can be ascribed to probiotics as a general class [3]. Several species of the genera *Bifidobacterium* and *Lactobacillus* are claimed to have a core benefit on healthy gut microbiota by creating a favorable gut environment. High quality meta-analyses also suggest that they are effective against infectious diarrhea, antibiotic-associated diarrhea, travelers’ diarrhea, slow gut transit, irritable bowel syndrome, abdominal pain and bloating, and ulcerative colitis [3,5,6,7,8,9,10,11,12,13]. In addition, strain-specific probiotics support positive health outcomes including the maintenance of a healthy immune system [3,14,15]. Despite a widely accepted definition and well-described benefits to health, the term probiotic is still misapplied by scientists and the general public as referring to fermented products that contain a diverse community of live, potentially beneficial microorganisms that are not specifically probiotics [3]. Probiotics are regarded as complementary and alternative medicine along with vitamins, minerals, and other dietary supplements [16]. Today, probiotics are commercially available substances that are found not only in dietary supplements, drugs, functional foods, and beverages, but also in products such as skin creams, vaginal capsules and tampons, and chewable tablets for gum health [17].

According to the ninth edition of the European Pharmacopoeia (2019) [18], ‘live biotherapeutic products’ for human use are medicinal products containing live microorganisms that can be administered orally or vaginally in different pharmaceutical forms. New terms such as ‘paraprobiotic’ or ‘postbiotic’ have emerged to denote that non-viable microbial cells, microbial fractions, or cell lysates might also offer physiological benefits to the host by providing additional bioactivity [19]. On the other hand, ‘prebiotics’ are substrates that are selectively used by host microorganisms conferring a health benefit [20], and ‘synbiotics’ are dietary supplements or food ingredients that combine the effects of probiotics and prebiotics [21].

Despite the widespread and easily accessible evidence that supports the benefits of probiotic use, health professionals may hesitate in recommending probiotics to patients when they receive conflicting messages. Health professionals may have difficulties in processing large volumes of information generated by commercial enterprises about the benefits and use of probiotics. Based on Regulation No. 1924/2006 of the European Union on nutrition and health claims made on foods [18], EU member states have forbidden the use of the term ‘probiotic’ on all foods including probiotic food supplements unless the procedures noted in the regulation have been observed. This can lead to considerable confusion among health professionals when advising patients on the use of probiotics. Therefore, our aim was to investigate the current knowledge, attitude, and practice of health professionals regarding probiotics.

## 2. Methods

The online survey was distributed via email and social media platforms using snowball sampling and was open for three months between March and May 2018. The English version of the online questionnaire was translated into six languages (Croatian, German, Hungarian, Italian, Slovenian, and Swedish). Participants were free to respond in the language of their choice.

The questions were modeled based on those used in previously published studies on the knowledge of probiotics [22,23,24,25,26]. The survey was divided into three sections. The first section consisted of demographic questions and the second section consisted of questions about the respondents’ knowledge of probiotics. In Section 2, the respondents were first asked to evaluate their knowledge of probiotics on a 5-point Likert scale with the following grades: no knowledge (1), little knowledge (2), medium knowledge (3), good knowledge (4), and very good knowledge (5). They were then asked about the definition of and facts about probiotics. The third section included questions about the respondents’ experience in the use of probiotics, their beliefs about the positive effects of probiotics, and whether they have ever advised the use of probiotics to friends, relatives, or patients. At the end of Section 3, the respondents were asked about their major sources of information regarding probiotics and if they would like to develop more extensive knowledge on probiotics. The survey took seven minutes on average to complete.

Ethics approval was obtained through the Ethics Committee for the field of nursing at the University of Maribor, Faculty of Health Sciences (study number: 038/2017/5755-3/501). The Human Research Ethics Committee of Western Sydney University also approved this research (RH12553). 

The data were analyzed by the IBM SPSS V25 software and MS Excel. A p value of less than 0.05 was accepted as statistically significant. Proportions were calculated and percentages reported for categorical variables. Proportions were compared by using the χ^2^ statistic. The numerical variables were presented as means ± standard deviation (SD). For variables that are not normally distributed, a median with a 95% confidence interval was used. The Mann–Whitney *U* test and its extension, the Kruskal–Wallis *H* test, were used to compare the self-evaluation of the knowledge of probiotics by sex and among different groups of health professionals.

## 3. Public Involvement

The public were involved in the research by answering the online survey. The survey was anonymous and voluntary. The respondents were given the opportunity to add their email address if they wished to receive a copy of the results.

## 4. Results

Health professionals from 30 countries (n = 1066) responded to this survey. Respondents were drawn from the following regions: Western Europe (n = 695; 65.2%), Central and Eastern Europe (n = 262; 24.6%), Australia (n = 96; 9.0%), and other (n = 13; 1.2%). Mean age ± SD of respondents was 47 ± 14 years, ranging from 19 to 78 years. In total, 859 (80.6%) of respondents were female and 207 (19.4%) were male. More than one third (35.7%) of the respondents had higher university degrees (master’s or PhD), 22.4% had a university degree, 13.6% completed higher education, and 25.1% completed secondary school. A small proportion of respondents (3.2%) did not reveal their education level.

Registered nurses or midwives represented the highest proportion of respondents from a single profession (25.3%), followed by pharmacists (16.7%), medical doctors or dentists (16.7%), allied health professionals (12.9%), psychologists (1.8%), and other health professionals including physiotherapists, paramedics, and osteopaths (27.3%). Most respondents had between 11 and 30 years of experience working as a health professional (47.8%), followed by those who had 10 years’ experience or less (33.9%), and those who had over 30 years of experience (18.2%).

### 4.1. Respondents’ Self-Evaluation of Knowledge of Probiotics

The respondents graded their knowledge of probiotics using the 5-point Likert scale according to five possible grades (1: No knowledge; 2: Little knowledge; 3: Medium knowledge; 4: Good knowledge; and 5: Very good knowledge). Most evaluated their knowledge as medium (36.4%) or good (36.2%). Some rated their knowledge as excellent (8.9%), some as poor (16.7%), and the remainder had no knowledge of probiotics at all (1.8%).

**Figure 1:**
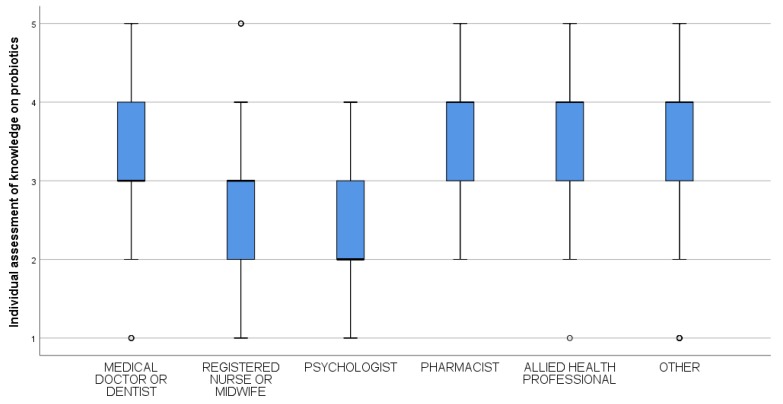
Self-evaluation of knowledge about probiotics by profession (1: No knowledge; 2: Little knowledge; 3: Medium knowledge; 4: Good knowledge; and 5: Very good knowledge). The boxplots show the quartiles for each group of healthcare professionals.

The self-evaluation of knowledge is statistically different among various groups of health professionals (H = 122.9; p < 0.001). Pharmacists, allied health professionals, and other health professionals evaluated their knowledge with a median grade of 4 (95% CI: [4,5]); medical doctors and dentists, registered nurses, and midwifes demonstrated a median grade of 3 (95% CI: [3,4]); and psychologists graded their probiotics-related knowledge with the lowest median value of 2 (95% CI: [2,3]) (Figure 1).

The median score for the self-evaluation of the knowledge of probiotics was 3 (95% CI: [3,4]) in both sexes, so there was no statistical difference between male and female health professionals in this respect (U = 83263.0; p = 0.134).

### 4.2. Respondents’ Knowledge of the Correct Definition of Probiotics

**Figure 2:**
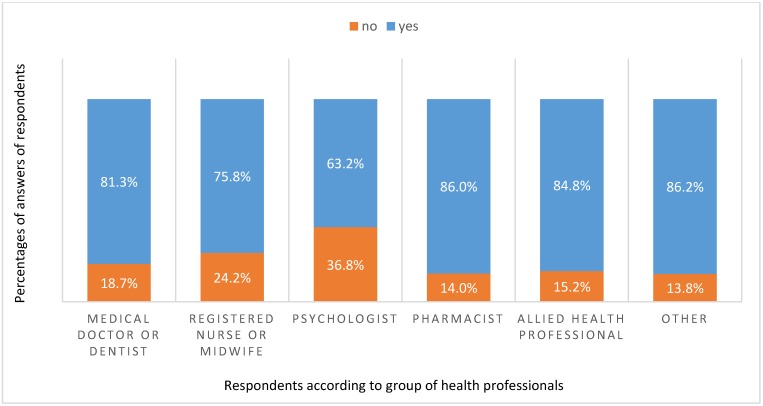
Knowledge of the correct definition of probiotics among different health profession groups. Blue proportions show the percentage of health professionals that chose the correct definition in each group.

The respondents then chose from five different claims for the definition of probiotics: (1) probiotics are dead microorganisms, that when administered in adequate amounts, confer a health benefit to the host; (2) probiotics are live microorganisms, that when administered in adequate amounts, confer a health benefit to the host; (3) probiotics are all microorganisms consumed with foods and dietary supplements; (4) probiotics are all microorganisms that adhere to intestinal epithelial mucosa; (5) I do not know the definition of probiotics. The correct definition (answer 2) was chosen by 82.2% of the respondents. The knowledge of the right definition of probiotics was statistically different among the various groups of health professionals (χ^2^ = 17.645; p = 0.003). Over 80% of pharmacists, allied health professionals, medical doctors and dentists, and other health professionals knew the correct definition of probiotics, whereas three quarters of registered nurses and midwives and less than two thirds of psychologists identified the correct definition (Figure 2).

When asked to choose the correct definition of probiotics, more female (83.6%) than male (76.8%) respondents chose the correct answer to a statistically significant degree (χ^2^ = 4.853; p = 0.028).

### 4.3. Respondents’ Knowledge of Microbial Species including Probiotic Strains

**Figure 3:**
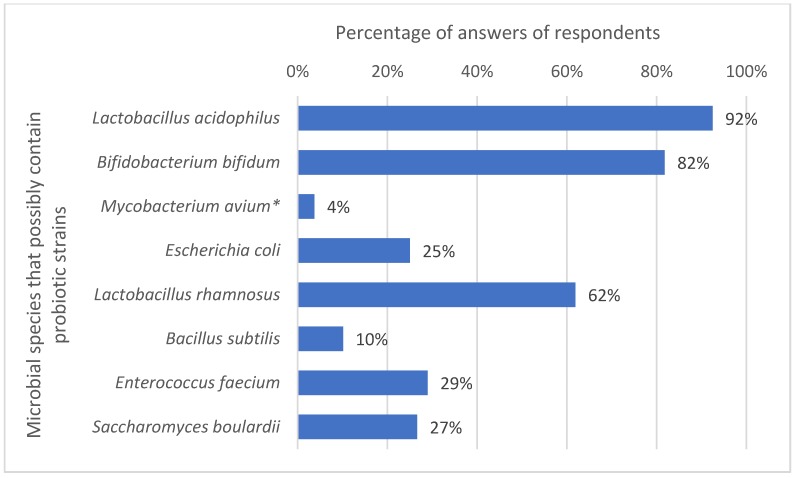
Respondents’ knowledge of microbial species that possibly have probiotic strains. * This species has no probiotic strains.

Respondents were then asked to choose species from the provided list of microorganisms that they thought had probiotic strains. The list included: *Lactobacillus acidophilus, Bifidobacterium bifidum, Mycobacterium avium, Escherichia coli, Lactobacillus rhamnosus, Bacillus subtilis, Enterococcus faecium*, and *Saccharomyces boulardii*. All mentioned species except for *Mycobacterium avium* contain different probiotic strains. As shown in Figure 3, *Lactobacillus acidophilus* (92%), *Bifidobacterium bifidum* (82%), and *Lactobacillus rhamnosus* (62%) were the most recognizable species of bacteria containing probiotic strains. A little less than a third of the respondents also chose *Enterococcus faecium* (29%) and *Saccharomyces boulardii* (27%) and, indeed, both species include several probiotic strains even though they are not so commonly known. One quarter (25%) also correctly listed *Escherichia coli*, which has one commonly known probiotic strain, *E. coli* Nissle 1917 [27]. The only species in the list without probiotic strains was *Mycobacterium avium*, which was incorrectly selected by a minority of respondents (4%).

**Figure 4:**
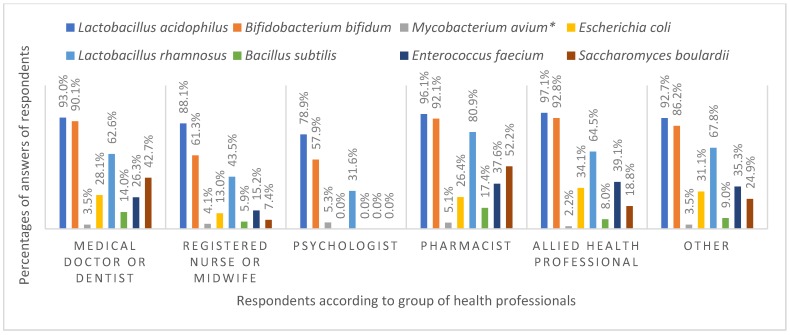
Respondents’ knowledge of microbial species with possible probiotic strains according to health profession. * This species has no probiotic strains.

The respondents’ knowledge about probiotic strains also differed considerably among the various groups of health professionals (Figure 4). *Lactobacillus acidophilus* was the most recognizable microbial species with probiotic strains among all of the health professional groups. The vast majority (>0%) of health professionals in all groups except registered nurses, midwifes, and psychologists recognized this species as including probiotic strains. The probiotic strains of *Bifidobacterium bifidum* were also widely identified, with more than 90% of medical doctors and dentists, pharmacists, and allied health professionals recognizing them. In contrast, less than two thirds of nurses and midwives (61.3%) and psychologists (57.9%) had heard of the probiotic strains of *Bifidobacterium bifidum*. *Mycobacterium avium* does not contain any probiotic strains and is an opportunistic pathogen, and was well known among all surveyed health professionals [28]. *Lactobacillus rhamnosus* is a somewhat less well-known species containing probiotic strains. However, this was recognized as a species containing probiotic strains by a majority of pharmacists (80.1%). *Escherichia coli* is another important bacterial species that has at least one probiotic strain, the above-mentioned *E. coli* Nissle 1917; however, approximately two thirds of allied health professionals (65.9%) and other health professionals (68.9%) were unaware of this. Similarly, the probiotic properties of certain *Enterococcus faecium* strains were known among all health professional groups to a small extent; only one third of pharmacists, allied health professionals, and other health professionals acknowledged that this species could contain probiotic strains. *Saccharomyces boulardii* was recognized as a yeast species with probiotic strains by some pharmacists and medical doctors (52.2% and 42.7%, respectively).

The familiarity with species of microbes that contained probiotic strains was statistically different by sex only for the fungi *Saccharomyces boulardii* (χ^2^ = 21.650; p < 0.001). Only 23.6% of female and 39.1% of male respondents knew that *Saccharomyces boulardii* contained probiotic strains.

### 4.4. Respondents’ Knowledge of some of the Claims for Probiotics

The respondents were asked to mark ‘true’ or ‘false’ for the following claims: (1) The only probiotics that work are tablets, powders, or capsules; (2) Probiotics need to be live microorganisms; (3) For a beneficial effect, it is necessary to consume probiotics for a long period of time as they disappear from the gut after two weeks; and (4) Probiotics should be taken before a meal.

Fewer than one quarter of the respondents (22.5%) incorrectly answered that probiotics are only effective in the form of tablets, powders, or capsules. Almost three quarters (73.7%) of them correctly answered that probiotics must be live microorganisms. Most respondents (71.5%) agreed that probiotics needed to be consumed orally for a long period of time to exert a beneficial health effect, as they may disappear from the gut after two weeks. Nearly two thirds of the respondents (64%) claimed that probiotics should be taken before meals. This claim was noted as correct in accordance with [29], wherein the highest survival of probiotics was observed when they were given before a meal, although this issue remains debatable.

Knowledge based on all statements differed significantly. Most of the respondents knew that efficient probiotics were not restricted to tablets, powders, and capsules. Only around 10% of registered nurses, midwifes, and psychologists marked this statement incorrectly as being true. The group that incorrectly identified this statement as being true in the highest percentage (36.2%) were allied health professionals.

More than 70% of all respondents from each group, except for registered nurses and midwives, knew that it is necessary to consume probiotics for a long period of time in order to maximize their beneficial effects. Knowledge about when best to take probiotics differed significantly between professional groups (χ^2^ = 28.375; p < 0.001). Over three quarters of pharmacists (77.5%) and more than two thirds of allied health professionals (71.0%) and psychologists (68.3%) recommended taking probiotics before meals, whereas medical doctors, dentists, nurses, and midwives had a more divided opinion about this. There was no statistical difference between sexes in familiarity with statements regarding probiotic properties, as 83.4% of the female respondents and 76.8% of the male respondents were familiar with these statements.

### 4.5. Respondents’ Use of Probiotics

**Figure 5:**
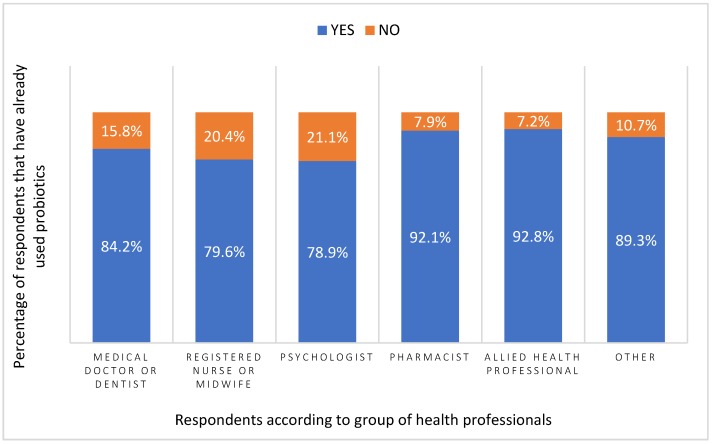
Use of probiotics among health professionals.

The vast majority of respondents (86.8%) had already used probiotics. Over 92% of pharmacists and allied health professionals had previously taken probiotics, followed by other health professionals (89.3%). More than 84% of medical doctors and dentists had already used probiotics as had almost 80% of registered nurses, midwives, and psychologists (Figure 5).

The respondents reported personally having consumed probiotic drinks or probiotic yogurts (74.5%), probiotic medicines (73.9%), probiotic food supplements available only in pharmacies (74.4%), probiotic food supplements available in specialized shops, supermarkets, or online (74.4%), and other probiotic products (34.1%). Other probiotic products that respondents claimed to have consumed included chewable tablets with probiotics for dental health, tampons and/or vaginal creams or capsules with probiotics, and skin creams with probiotics. More female (88.2%) than male (80.7%) respondents had previously used probiotics. The difference was statistically significant (χ^2^ = 8.319; p = 0.004).

### 4.6. Reasons for Taking Probiotics

More than half of the respondents (53.0%) had taken probiotic products for preventive purposes during an antibiotic treatment (44.5%), for improved digestion (36.8%), to reduce bloating and also for other reasons such as allergic conditions, dermatological problems, or stress (20.2%).

Most respondents believed that probiotics had beneficial effects if taken during antibiotic therapy (90.2%). Most agreed that probiotics should be used in the case of diarrhea (83.5%), for constipation (70.6%), and before traveling abroad (63.3%). Most also believed that probiotics were beneficial in treating allergies (60.4%). More than half of the respondents also thought that taking probiotics could be good for people with depression or mood disorders. A little less than 50% believed that probiotics were effective against hay fever, carcinoma, and liver disease.

### 4.7. Sources of Information on Probiotics

Respondents had acquired their knowledge of probiotics from various sources including books or expert magazines (53.3%), websites (34.9%), at work (28%), from pharmacies (25%), and from radio or TV (9.7%). More than half of all respondents (57.5%) wanted to learn more about probiotics.

### 4.8. Advising the Use of Probiotics

A total of 933 health professionals (87.5% of all respondents) had advised people such as friends (67.6%), relatives (63.8%), sons and daughters (43.6%), and others including clients, co-workers, visitors or patients to use probiotics.

**Figure 6:**
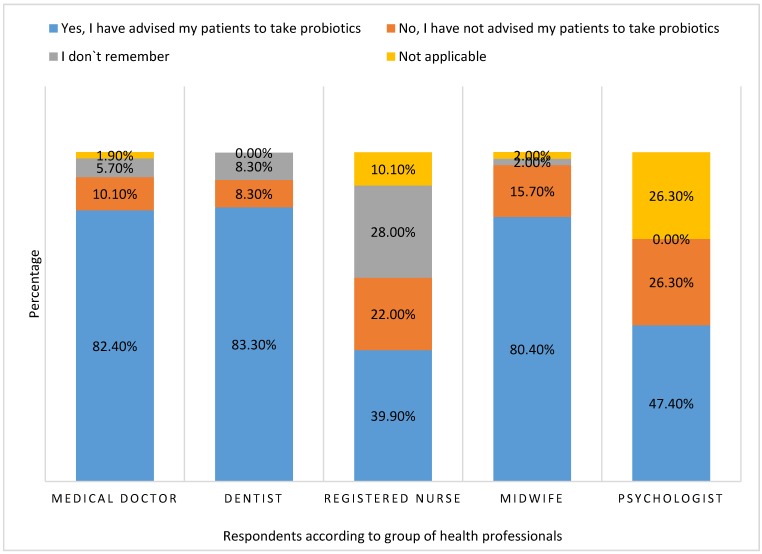
Advice regarding the use of probiotics to patients by profession.

Respondents were also asked if they had ever advised their patients to use probiotics, and almost 79% of them answered affirmatively, whereas 5.3% of the respondents did not remember ever doing so. Only 11.6% of healthcare workers had never advised a patient to try probiotics. We also analyzed this question separately for medical doctors, dentists, registered nurses, midwives, and psychologists (Figure 6). Unlike registered nurses and psychologists, the majority (>80%) of medical doctors, dentists, and midwives had advised their patients to use probiotics.

### 4.9. Respondents’ Knowledge of Prescription Probiotics

**Figure 7:**
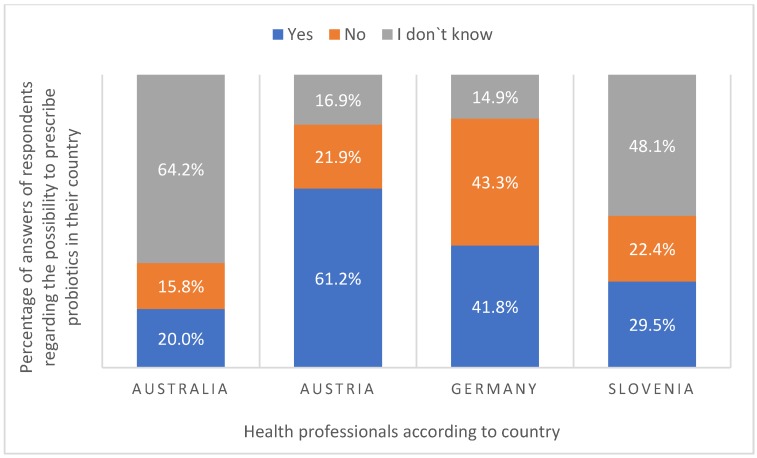
Respondents’ knowledge of prescription probiotics by country.

Knowledge about the prescription of probiotics varied between respondents from different countries. Some respondents believed that it was possible to prescribe probiotics in their country (41.6%), while others believed that it was not possible (29.5%), and some others admitted that they were not sure if it was possible or not (28.9%). As different countries have different regulations regarding prescription, we analyzed those with the highest numbers of respondents (Figure 7).

In all four noted countries (Australia, Austria, Germany, and Slovenia) it is not possible to prescribe probiotics. Only 22.4%, 21.9%, and 15.8% of Slovenian, Austrian, and Australian health professionals, respectively, answered this question correctly. In contrast, over 40% of German health professionals correctly answered that it was not possible to prescribe probiotics in Germany.

## 5. Discussion

Here, we present a cross-sectional study of the knowledge and beliefs of healthcare professionals with regard to probiotics. One important limitation of our work was the number of respondents that completed the survey. The number of clicks on the survey was much higher than the actual number of completed questionnaires (n = 1066). This may have been because some healthcare professionals did not feel that they had enough knowledge to correctly answer the survey questions or perhaps some of them did not agree with the questions, therefore, it would be much more likely for someone who knows about probiotics to respond than someone who knows little to nothing. Due to anonymity, we had no information as to who was responding to the survey. Additionally, there was a disconnection between the people’s perception of how good their knowledge of probiotics was and their ability to answer the questions correctly. Another limitation was the high female-to-male ratio of approximately 4:1. As a result, our sampling was not statistically validated.

In our study, a statistically significant difference was found in the respondents’ self-evaluation of their knowledge of probiotics among the various health profession groups (H = 122.9; p < 0.001). Pharmacists, allied health professionals, and other health professionals graded their knowledge as good (4), whereas medical doctors and nurses evaluated their knowledge with a lower grade (3). The medical doctors in our study were more self-critical than those surveyed by Johnson et al. [22], who found that 85% of doctors in the study group had a good knowledge of probiotics. However, there was no difference in the self-evaluation of nurses involved in this study. Similar to our findings, the majority (62.9%) of practice nurses questioned by Johnson et al. [22] believed that they had an average understanding of probiotics.

Although there is a large body of scientific evidence supporting the benefits of probiotics, the wide range of probiotic products and certain strain-specific effects of probiotics have made it difficult for health professionals to make evidence-based decisions regarding probiotics [30]. The findings from our study tend to support this contention as medical doctors and nurses did not evaluate their knowledge with the highest score. In another study, pediatricians evaluated their knowledge as quite high [31]. The pressure on nurses to broaden their skill set and to take on more responsibility for patient care has led to a need for nurses to specialize in various areas of health care. Patients may also find it increasingly difficult to obtain reliable information on probiotics and may therefore ask questions when visiting their nurse. While general practitioners are more likely to prescribe probiotics to patients, specialist gastrointestinal and stoma care nurses should be aware of the potential benefits of probiotics and how they work. People with conditions such as inflammatory bowel disease may ask specialist nurses for advice on the potential of probiotics to improve their condition, and these secondary care professionals should have easy access to reliable information based on the latest evidence regarding particular probiotics that patients could consider using [22].

Our study also revealed that the majority (82.2%) of respondents correctly identified the definition of probiotics and that knowledge of the correct term was statistically different among the various health profession groups (χ^2^ = 17.645; p = 0.003). More than 80% of pharmacists, allied health professionals, medical doctors, dentists, and other health professionals identified the correct definition of probiotics, whereas the corresponding percentage for registered nurses and midwives was 75. In the study by Soni et al. [23], the knowledge of probiotics among health students of various disciplines also differed significantly, with approximately 93% of participants being aware of the term ‘probiotics’ and about 80% of them defining it correctly. A similar study in [32] also reported a low level of knowledge of probiotics among university students and emphasized the need for education about the nutritional and health benefits of fermented dairy products, especially those containing probiotics.

Regardless of the professional group, over 70% of our respondents correctly answered that probiotics had to be live microorganisms. This was a higher proportion than that reported in another study [24], where 65.6% of the health professionals stated that probiotics contained live microorganisms, with the highest percentage of correct answers coming from pharmacists and physicians. Only 18.6% of nurses correctly identified that probiotics were microorganisms. On the other hand, 14.5% of the respondents in that study incorrectly stated that probiotics were synthetic drugs and 14.9% stated that probiotics were natural plant products. These misperceptions could be corrected with the implementation of educational interventions potentially delivered over a long period of time to ensure the ongoing retention of information. However, this is not easy because health claims for probiotics in the EU are not allowed to be explained due to current nutrition and health-claim legislation in EU member states, in fact even the term ‘probiotic’ is forbidden [30]. For this reason, health professionals should be able to access scientific information directly from probiotic companies, as many probiotic companies have health professional-specific websites through which scientific information and educational publications are available. In addition, as an indication of product quality, the product label should show the full name of the probiotic strain(s) as well as the minimum live count of the probiotic strains throughout the shelf-life of the products [22].

Some previously published studies [24,25,33] have assessed the knowledge, awareness, and perception of probiotics among health providers in Nigeria. The results of these studies revealed a low level of familiarity by clinicians and health professionals with probiotics. Conversely, other studies conducted in the USA, Europe, and Asia [26,34] have reported a high degree of familiarity with the term ‘probiotics’ among health professionals. This may be because probiotic foods are reasonably well-established in North America, Europe, and Asia in comparison to Africa, and companies manufacturing probiotic products in North America, Europe, and Japan have generally not made probiotic products available in Africa, probably due to the low market power of the population [33,35]. Medical students in Nigeria [24] have the highest level of knowledge of probiotics, followed by pharmacy students, which may be due to the high quality of university instruction and the use of a growing volume of evidence that supports the health effects of probiotics. Several studies have also emphasized that health providers clearly need more information about probiotics in order to confidently provide advice to patients [24,36]. In particular, nurses have a crucial role supporting the use of probiotics because they are involved in the routine care of patients as well as in providing advice and education [22].

We found a statistically significant difference in the knowledge of the correct definition of probiotics in favor of females (χ^2^ = 4.853; p = 0.028). Other studies have reported different results, ranging from non-significant differences [23,34] to male participants having a higher knowledge [37].

Correct information about probiotics is also important for patients because their main source of information is commercial advertising rather than healthcare professionals [38]. Several studies have shown that patients and other consumers have limited knowledge about probiotics [39,40,41]. Although general practitioners, nurses, and other health professionals may sometimes advise patients to take probiotics [22], they can also freely purchase these products in pharmacies, specialty shops, supermarkets, and online for whatever purpose they choose.

Some health professionals are confused about yoghurts, cultured milks, and fermented foods because they do not understand that such products do not necessarily contain probiotics, whereas others believe that probiotics only work in the form of tablets, powders, or capsules [22]. Several health professionals think that foods containing probiotics are a better way to ingest probiotics than probiotic drugs because of the synergistic effects between food components and probiotics and because food enhances the stability of consumed probiotics [23]. There is a growing body of scientific evidence showing the beneficial health effects of probiotic fermented dairy foods on a wide variety of diseases including gut disorders (e.g., irritable bowel syndrome, constipation, and diarrhea), infections (e.g., antibiotic-associated diarrhea, *Clostridium difficile*, and *Helicobacter pylori*) as well as metabolic disorders and cancer [22,42,43,44,45,46,47,48,49]. The mechanism of action most commonly attributed to probiotic microorganisms is the production of antimicrobial substances, followed by competitive exclusion and competition for essential nutrients [37].

The probiotic strains belonging to the *Lactobacillus* and *Bifidobacterium* genera that are most commonly used as probiotics are well known [3,4,19]. Some of the respondents also recognized gram-positive *Enterococcus faecium* and gram-negative *Escherichia coli.* As both species contain strains that are natural inhabitants of the gastrointestinal tract, it is understandable that some of their strains are probiotic such as *Enterococcus faecium* PTA 5844 and *Escherichia coli* Nissle 1917 [50,51,52]. However, both species contain several pathogenic strains such as vancomycin-resistant *Enterococcus faecium* or enteropathogenic, enterohemorrhagic, and enterotoxigenic *Escherichia coli* as well as *Escherichia coli* strains resistant to extended-spectrum beta-lactamases [27,53,54] that can cause severe diseases. The only yeast species mentioned in our survey that contains probiotic strains, *Saccharomyces boulardii*, was recognized in a lower percentage by the surveyed health professionals, mainly by the surveyed pharmacists and medical doctors, and recognized by more male than female respondents. On the other hand, a few respondents added *M. avium* as a species that also contained probiotic strains, even though *M. avium* is an opportunistic pathogen, mainly in animals, and no probiotic strains are known [28].

Most of our respondents (64%) claimed that probiotics should be taken before meals. Commercial literature on various probiotic products suggests that they can be taken before meals, during meals, after meals, or even without meals [29]. This has led to confusion amongst both health professionals and consumers. To our knowledge, there is only one scientific study that has addressed this question. The authors in [29] confirmed the highest survival of non-enteric coated probiotics if given with a meal or before a meal, and the lowest survival if taken after a meal. In theory, the buffering of stomach pH by certain foods should improve the gastric survival of probiotic microorganisms, but to what extent remains unknown [55,56]. The protective matrix of fat in milk and dairy products also has a positive effect on probiotic viability [55]. One study found higher survival rates for probiotic microbes as components of foods and in various micro- or nano-encapsulation matrices [57]. Another study found a higher survival of probiotics in a yogurt matrix and capsules [56]; however, another efficiently used the rehydration of powdered solution [58] and another concluded that foods were better carriers for probiotics than supplements [59]. In general, these studies show that there are many efficient possibilities and that more studies are necessary to make correct claims. As a rule, each formulation should be tested in a clinical study that takes into account the time of application.

Our results have also shown that health professionals mostly purchase probiotics in pharmacies for their own consumption because a free consultation is offered there. The pharmacists in our study demonstrated a high level of knowledge of probiotics including the specific strains and their mechanisms of actions, which is understandable given that most probiotic products are sold over-the-counter in their pharmacies and that they need to be up to date in order to be able to consult their patients [60,61].

In Slovenia, it is not possible to prescribe probiotics. Over-the-counter probiotic food supplements can only be recommended. A very limited amount of probiotic drugs that have been manufactured in Slovenia, are part of the Slovenian drug register [62,63]. In Australia, probiotics occupy a liminal space between medicine and food. Probiotics are regulated as a food when the product makes no specific claims of health benefit, but it is regulated as a “therapeutic good” if it does. There are 245 probiotic products listed on the Australian Register of Therapeutic Goods (ARTG), all of which can be purchased commercially without a prescription from a medical practitioner [64]. In Austria and Germany, probiotics are regarded as nutrients and form the generic term for dietary supplements and dietetic foods. The boundary between the nutritional or physiological effects of food supplements (as food) and pharmacological action of medicinal products is important for their authorization, notification, registration, and labeling. Products authorized as medicinal products cannot be sold as foods, and foods cannot be sold as medicinal products. Probiotic supplements have a nutritional or physiological effect, while medicines are used to cure, alleviate, or prevent human diseases. In Austria, a delimitation advisory committee has been set up with the Federal Ministry for the preparation of reports on the delimitation of medicines from foods. Probiotics can be recommended by health professionals, whereas probiotic supplements (as food) are freely available [65]. Almost four fifths (79%) of our respondents, mainly medical doctors, dentists and midwives, had prescribed or advised their patients to use probiotics. Other authors have reported lower values ranging from 25.8% [24] to 50% [23] and 65.5% [22,30]. In comparison, 63.8% of the respondents to our survey stated that they prescribed or recommended the use of antibiotics regularly [24].

The main reasons the respondents in our study advised patients on the use of probiotics included a course of antibiotics, diarrhea, constipation, and traveling abroad. A total of 60% of our respondents also believed that probiotics were effective against allergies, depression, or mood disorders. Further reasons why health practitioners in other studies advised the use of probiotics include irritable bowel symptoms, susceptibility to common infections, risk of diverticulitis, ulcerative colitis, old age, and mothers or babies in families with a tendency to allergy [22,23,30].

## 6. Conclusions

Several factors such as increasing antibiotic resistance among pathogenic bacteria as well as the increased demands of consumers for natural substitutes for medicines have led health professionals to explore alternatives to pharmaceutical drugs. The emergence of scientific and clinical evidence showing the effectiveness of certain probiotic strains has made them attractive as biotherapeutics. As the current EU legislation on nutrition and health claims prevents manufacturers from explaining their probiotic products to the consumers [30], the role of health professionals in giving informed and objective advice on probiotics has largely increased. Patients who are interested in probiotics may have difficulties finding reliable information on what probiotics do and how they actually work, and may ask questions about probiotics when they visit their doctor’s office. Therefore, it is important that health practitioners receive scientific evidence-based advice so that patients receive objective and informed advice from their general practitioner, nurse, or pharmacist, rather than obtaining questionable information from unreliable sources [30]. As health practitioners have indicated an interest in knowing more about probiotics [25], and are also the ones responsible for preparing various guidelines for probiotic use based on evidence-based research [66], incorporation of this topic in the curriculums of future health professionals and targeted learning programs of current healthcare professionals may help in improving their knowledge and awareness.
